# Supplementary material for: Pharmacoscreening, molecular dynamics, and quantum mechanics of inermin from Panax ginseng: a crucial molecule inhibiting exosomal protein target associated with coronary artery disease progression
Source: PeerJ. 2023 Dec 6;11:e16481. doi: 10.7717/peerj.16481 (PMC10710165; doi:10.7717/peerj.16481)
Supplement: Supplemental Information 1 [file peerj-11-16481-s001.docx]

**Figure S1:** Gene ontology analysis. (A) Biological process, (B) Molecular function, and (C) Cellular components.


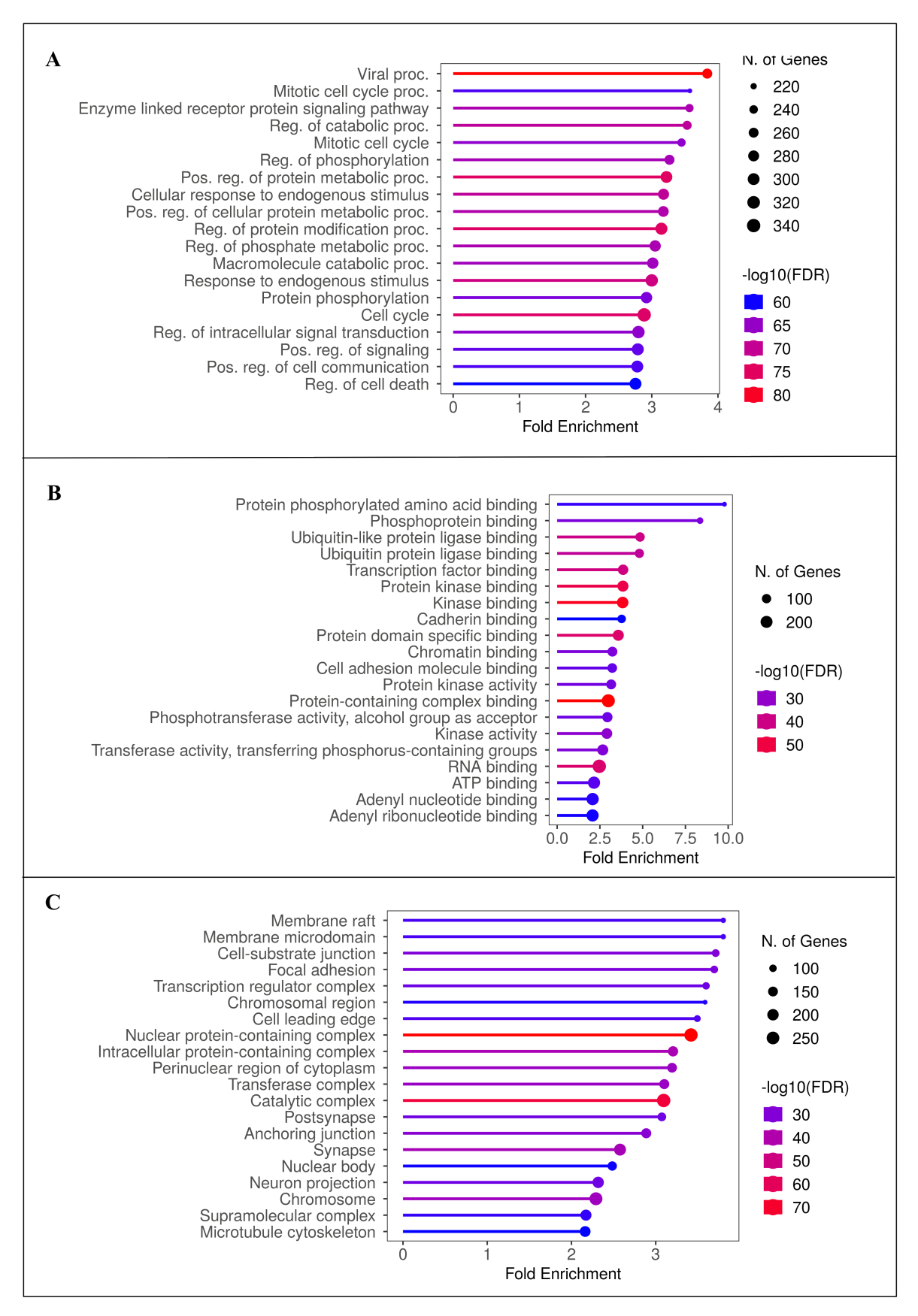


**Figure S2:** KEGG pathway analysis


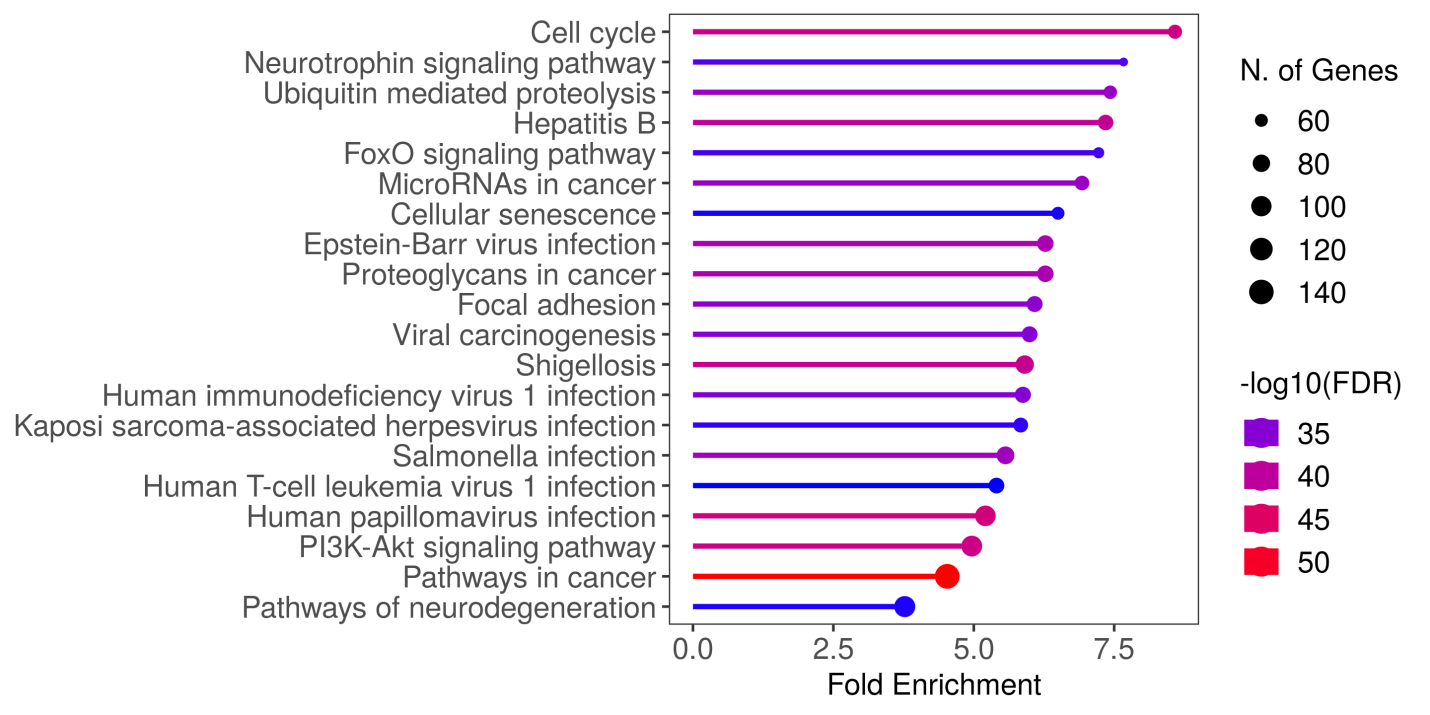
.
